# *Preventing Chronic Disease*: Celebrating Contributions and Defining Future Directions

**DOI:** 10.5888/pcd14.170013

**Published:** 2017-02-02

**Authors:** Leonard Jack

**Figure:**
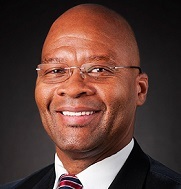
Leonard Jack, Jr, PhD, MSc, Editor in Chief

It is an honor to serve as the third editor in chief of this globally recognized journal, which now reaches more than 70,000 subscribers, and a pleasure to provide the first of a series of communications for the editor in chief’s column. This column will be published 3 times annually in February, June, and October. I will provide updates on the journal’s progress, comment on timely public health topics, make announcements and acknowledgments, and raise issues of interest to *Preventing Chronic Disease *(*PCD*) readers. Exciting days lie ahead for *PCD*, and we look forward to providing our readers with access to relevant, rigorous research and evaluation findings that can benefit public health practice. As a result of strong leadership, support from the Centers for Disease Control and Prevention’s (CDC’s) National Center for Chronic Disease Prevention and Health Promotion (NCCDPHP), and the unwavering commitment to excellence from a team of *PCD* staff members, the journal is well-positioned for an exceptional future.

## *PCD* has a new home

*PCD* has a new home in the Office of Medicine and Science (OMS). In October 2016, NCCDPHP announced the establishment of OMS under the leadership of Dr Peter Briss. OMS brings together the Office of the Associate Director for Science, the Office of the Medical Director, and *PCD*. OMS has 3 primary goals: 1) enhance the conduct, quality, communication, and impact of public health science, 2) establish or strengthen linkages between health care and public health, and 3) provide service to NCCDPHP and CDC on topics related to science and medicine. *PCD*’s placement in OMS will help advance the journal’s mission to promote the open exchange of information and knowledge among researchers, practitioners, and others who strive to improve the health of the public through chronic disease prevention.

## Special thanks and acknowledgments

A special thanks is extended to Dr Samuel Posner, who provided 7 years of dedicated service to *PCD* as its second editor in chief. Dr Posner now serves as Associate Director for Epidemiologic Science at CDC’s National Center for Immunization and Respiratory Diseases. His legacy is one of scientific excellence, technical innovation, and service. It was his initiative to transition *PCD* to continuous publication in 2012 to reduce production turnaround. When *PCD* presented data at the Council of Science Editors annual meeting in 2014 on the journal’s experience in designing and implementing a continuous workflow and showed that *PCD* had reduced the time from acceptance to publication by 43% (220 days in 2011 to 126 days in 2013), it was to a standing-room–only audience. Dr Posner’s passion for innovation extended to social media and outreach as well, and during his tenure *PCD* developed apps for iPhone, iPad, and Android devices; established a Facebook page; collaborated with Medscape to offer continuing medical education credits; and pursued other tools and resources to expand *PCD*’s reach. To improve the journal’s editorial quality and service to the public health community, Dr Posner created a team of associate editors to review manuscripts, including an associate editor for the new GIS Snapshots, which take advantage of *PCD*’s online format to display large-scale maps of key health indicators. He recognized the value of mentoring students on the importance of publication and established *PCD*’s Student Research Paper Contest, which has exposed a new generation of public health practitioners to *PCD*. Under Dr Posner’s leadership, *PCD* was named a Google Scholar top-20 journal in public health and increased its Thomson Reuters impact factor to be competitive with other prestigious journals in chronic disease prevention and health promotion. We are grateful to Dr Posner for his leadership, dedication, and service. Because of his legacy, *PCD* is positioned as a valuable resource to public health professionals around the world.

It is important also to acknowledge Dr Richard Goodman, another public health scholar, who worked closely with Dr Posner to elevate *PCD* as a valued resource in the global public health community. Dr Goodman was the first associate editor at *PCD*. He was instrumental in increasing the number of Original Research manuscripts that were peer reviewed. Dr Goodman’s broad expertise in medicine, public health, and law enhanced the quality and range of content published in *PCD*. Thanks to him, *PCD* published a series of articles on public health law, the first of which appeared in early 2004. Dr Goodman was a tireless champion for *PCD* and worked closely with countless authors during the editorial and peer-review process to ensure that the best and most useful science was published. He consistently asked authors to state explicitly what actions could be undertaken as a result of their research to improve health outcomes, to make sure *PCD* was a journal of public health action, not public health contemplation. Dr Goodman served CDC and the public with dedication and commitment. He retired from CDC in 2016, and we wish him the best in his future endeavors.

## Ten new* PCD* priorities

Since joining *PCD* as editor in chief in October 2016, I have established 10 priorities: adopting a multilayered approach to peer review, securing additional associate editors, promoting global public health perspectives, ensuring scientific integrity, publishing collections of articles, accelerating the dissemination of critical research, introducing a new article type to focus on implementation evaluation, maintaining the Student Paper Research Contest, developing tools and resources for novice authors, and enhancing our brand as an innovator in scholarly publishing.

### Adopting a multilayered approach to peer review

*PCD* now uses a multilayered approach to reviewing and approving manuscripts. This new approach has strengthened *PCD*’s ability to identify the highest-quality manuscripts. Each submission receives an initial internal review to determine whether it aligns with the mission and vision of *PCD* and meets minimal submission and context requirements for its article type. Manuscripts that meet initial screening expectations are then assigned to an associate editor. Thereafter, the associate editor provides the editor in chief with a recommendation based on his or her own assessment and feedback from peer reviewers. Final decisions are made by the editor in chief and communicated to authors.

### Securing additional associate editors

To meet the demands of an increasing number of submissions and an expanding scope of public health topics, I recruited 7 new associate editors. Collectively, they bring impressive expertise in population-level interventions, epidemiological studies, biostatistics, behavioral science, program evaluation, social determinants of health, health systems change and research, implementation science, and the dissemination of research and evaluation findings into public health practice. To learn more about *PCD*’s associate editors, you can visit www.cdc.gov/pcd/about_the_journal/associate_editors.htm.

### Promoting global public health perspectives

*PCD* is committed to its mission of serving as a resource to researchers and practitioners internationally. In today’s increasingly interconnected world, many factors that influence health in the United States are similar to factors that influence health in other countries. Many problems and challenges are similar, too. To respond to these global public health issues, *PCD* will focus even more than before on increasing the number of timely and relevant articles describing effective population-based interventions that address arthritis, asthma, cancer, depression, diabetes, obesity, cardiovascular disease, and other chronic diseases from a global health perspective. To this end, *PCD* has begun the process of recruiting subject matter experts in other countries to become associate editors or to join the journal’s editorial board.

### Ensuring scientific integrity

*PCD* revisited its protocol to ensure the scientific integrity of the information communicated in its articles. The journal has taken steps to provide associate editors, peer reviewers, and authors with essential guidance and information on what constitutes scientific misconduct. For information on *PCD*’s policy on scientific integrity, please visit www.cdc.gov/pcd/about_the_journal/editorial_policy.htm.

### Publishing collections of articles

*PCD* is often approached by authors seeking to publish a collection of manuscripts focused on a common theme. *PCD* has published many collections (eg, childhood obesity, multiple chronic conditions, veterans’ health) and will continue to do so. Authors interested in publishing a collection in *PCD* must first submit a proposal. In November 2016, *PCD* released new guidelines on submitting such proposals. You can learn more about previously published collections and submitting a proposal by visiting www.cdc.gov/pcd/collections/index.htm.

### Accelerating the dissemination of critical research

In fall 2016, *PCD* encouraged potential authors to submit Research Briefs through a call for manuscripts. A Research Brief, which has a text of 1,000 words or fewer, is a condensed version of an Original Research article, which typically has 3,000 words. The benefits of a Research Brief are that it moves faster through the peer review and editorial processes and thus abbreviates the time between submission and publication. Research Briefs, like Original Research articles, describe studies of interest to a broad audience and explain the value of the research to reducing or preventing chronic disease.

### Introducing a new article type to focus on implementation evaluation

Plans are being finalized for a new peer-reviewed article type, Implementation Evaluation. Implementation Evaluation articles will provide information for the real-world practice of public health and embrace the complexity under which public health interventions are implemented. The goal of providing this new article type is to advance the translation of evidence-based evaluation findings into public health practice by demonstrating outcomes that authentically document the value of chronic disease programs in improving the health of populations. Articles will share information on innovations in evaluation theory, planning, design, capacity, and tools and will account for the numerous and ever-present real-world factors, including program focus, population characteristics, staffing, health conditions of interest, geographic location, and human and fiscal resources, that shape rigorously conducted evaluation. Implementation Evaluation articles will examine various methods of evaluation conducted at various points in a program’s existence. Although *PCD* welcomes the submission of manuscripts describing any type of outcome evaluation — formative, process, short-term, and long-term — we will give priority to manuscripts reporting short-term and long-term outcome evaluation findings. In March 2017, we will post submission requirements for this new article type and announce a call for manuscripts.

### Maintaining the Student Research Paper Contest

*PCD* will continue its commitment to mentor future public health researchers and practitioners by sponsoring its annual Student Research Paper Contest. We have extended the deadline for submission from early January to March 10, 2017. *PCD* is looking for high school students, undergraduate and graduate students, medical residents, and recent postdoctoral fellows to submit manuscripts on prevention, screening, surveillance, and population-based interventions addressing chronic diseases. Winning manuscripts in each of these categories will be published in September 2017 with an accompanying editorial, student-focused podcast interviews, and social media promotion. Manuscripts must be received electronically no later than 5:00 PM EST on March 10, 2017. More information is available at www.cdc.gov/pcd/issues/2016/pdf/2017studentresearchpapercontestflyer.pdf.

### Developing tools and resources for novice authors

Not everyone has extensive experience in scientific and programmatic writing. Authors new to writing and submitting manuscripts for peer review can benefit from instruction, guidance, and encouragement. To provide assistance to these authors, *PCD* will create and publish tools and resources that novice authors can access to strengthen their skill set and build their confidence in scholarly writing.

### Enhancing our brand as an innovator

*PCD* received more than 600 manuscript submissions in 2016. We anticipate this number to rise in 2017, and we view this increase as an indication that the journal resonates with readers around the world. *PCD* will continue to evolve and innovate to meet the needs of its readership. The progress and growth of *PCD* would not have been possible without our contributing authors, reviewers, associate editors, editorial board members, the journal’s growing readership, and a highly capable staff with years of experience working diligently behind the scenes. In March 2017, *PCD* will engage its associate editors and editorial board members, NCCDPHP leadership, and external experts to identify ways to further enhance the journal’s content, reach, and global utility.

## Final thoughts

Thank you for a tremendously successful 2016. Through your support and contributions, *PCD* published articles on a variety of topics that our readership found informative and helpful.

Thousands of conversations about scholarly content take place online every day. Altmetric tracks a range of sources to capture, collate, and score this activity. *PCD* is pleased to share with our readership the top 10 articles published in 2016 as rated by Altmetric: www.cdc.gov/pcd/announcements/2016s-top-10-articles.htm. These articles address topics ranging from opportunities for physical activity in the workplace, improvements and disparities in types of foods and milk beverages offered in elementary school lunches, and the clustering of 5 health-related behaviors for chronic disease prevention among adults.

I look forward to providing *PCD* readership updates on website and mobile platform enhancements, announcements, appointments, and news on emerging public health topics. You can also stay current on recent publications and *PCD* developments by following us on Facebook and Twitter. The first major update on *PCD* activities will be described in the March 2017 release of our *2016 Year in Review*. Be sure to check back then to read about last year’s achievements; key metrics, such as the number of total submissions, rejection rate, peer reviews, and news and social media uptake; and additional information on our plans.

If you have an interest in submitting a manuscript to *PCD*, you can explore the journal’s website at www.cdc.gov/pcd. The website provides information on our various article types, topic areas of interest, submission requirements, and policies on scientific integrity. In 2017, *PCD* will continue to promote the global exchange of information and knowledge among researchers, practitioners, decision makers, and others who strive to improve the health of the public through effective chronic disease prevention and control. 

